# Effect of Endoplasmic Reticular Stress on Free Hemoglobin Metabolism and Liver Injury

**DOI:** 10.3390/ijms19071977

**Published:** 2018-07-06

**Authors:** Sung-Hui Tseng, Ting-Yun Chang, Chun-Kuang Shih, Rong-Hong Hsieh, Chia-Wen Chen, Yi-Chun Chen, Mei-Hsiang Lin, Jung-Su Chang

**Affiliations:** 1 Department of Physical Medicine and Rehabilitation, Taipei Medical University Hospital, Taipei 110, Taiwan; m003089010@tmu.edu.tw; 2 Department of Physical Medicine and Rehabilitation, School of Medicine, College of Medicine, Taipei Medical University, Taipei 110, Taiwan; 3 School of Nutrition and Health Sciences, College of Nutrition, Taipei Medical University, Taipei 110, Taiwan; soul0528@hotmail.com.tw (T.-Y.C.); ckshih@tmu.edu.tw (C.-K.S.); hsiehrh@tmu.edu.tw (R.-H.H.); d301091008@tmu.edu.tw (C.-W.C.); yichun@tmu.edu.tw (Y.-C.C.); 4 School of Food Safety, College of Nutrition, Taipei Medical University, Taipei 110, Taiwan; 5 Department of Pharmaceutical Science, School of Pharmacy, Taipei Medical University, Taipei 110, Taiwan; mhl00001@tmu.edu.tw; 6 Graduate Institute of Metabolism and Obesity Sciences, College of Nutrition, Taipei Medical University, Taipei 110, Taiwan

**Keywords:** liver injury, endoplasmic reticular stress, soluble (s) CD163, free hemoglobin

## Abstract

Elevated soluble (s) CD163 and free hemoglobin (Hb) levels predict fatty liver progression; however, the molecular mechanisms underlying Hb metabolism and liver injury remain undefined. We investigated the effects of endoplasmic reticular (ER) stress on red blood cell (RBC) rheology and free Hb recycling pathways. ER stress was induced in Sprague-Dawley rats by an intraperitoneal injection of tunicamycin (TM) (50, 100, and 200 μg/100 g body weight (BW)) or an intravenous injection of Hb (5 mg/100 g BW). A TM injection increased sCD163 levels, attenuated free Hb uptake, and maintained RBC aggregability. An Hb injection increased serum LVV-hemorphin-7 and total bilirubin levels, but this effect was suppressed by TM. A Western blot analysis showed that ER stress suppressed Hb degradation in the liver through downregulation of globin degradation proteins cathepsin D and glyoxalase-1, as well as heme degradation protein heme oxyganase-1 and keap-1 expression. An ER stress activator also increased the translocation of nuclear factor (NF)-κB (p65) and nuclear factor-erythroid 2-related factor 2 (Nrf2) to nuclei. In conclusion, ER stress triggers ineffective Hb metabolism via altering globin and heme iron degradation pathways. Inability to recycle and metabolize free Hb may underlie the association between iron dysfunction and liver injury.

## 1. Introduction

Non-alcoholic fatty liver disease (NAFLD) is the most common chronic liver disease worldwide [1]. Emerging evidence suggests that red blood cell (RBC)-CD163-hemoglobin (Hb) recycling pathways are associated with metabolic disorder such as NAFLD [2,3,4]. Free Hb levels in the serum predict NAFLD progression [2,3,4]. An elevated serum soluble (s) CD163 level is commonly observed in obese patients [5], as well as patients with metabolic disorder diseases [6,7,8]. Bariatric surgery [7,9] and lifestyle intervention significantly decrease sCD163 [10].

Macrophages are responsible for engulfing senescent or damaged RBCs [11,12]. Activation of Hb-degradation pathways also triggers an important antioxidative and cytoprotective response in the liver [13]. RBCs are synthesized in bone marrow and have an average life span of 120 days [11,12]. Macrophages take up senescent RBCs through erythrophagocytosis in the liver and spleen. Macrophages can also scavenge free Hb by CD163 surface receptors [12,13]. In normal physiological conditions, the free Hb, derived from intravascular hemolysis, is estimated to be 10–20% of the total turnover of RBCs [12]. Circulating free Hb, a highly reactive and unstable molecule, is first bound to haptoglobin (Hp) and then Hb-Hp complexes are taken up by CD163+ macrophages [12]. Inside macrophages, the globin moieties of Hb undergo proteolytic degradation by lysosomal proteolytic enzymes (e.g., cathepsin D (CTSD)) or cytosolic enzymes (glyoxalase I (GLO-1)) [14]. Heme iron is further degraded by heme oxygenase (HO)-1, and it is stored as the less-toxic Fe^3+^ in ferritin. This process also generates cytoprotective effector molecules such as biliverdin and carbon monoxide (CO) [15,16].

Accumulating evidence has shown that increased endoplasmic reticulmn (ER) stress-associated responses contribute to the pathophysiology of liver injury [1]. The ER is an organelle that plays a key role in nutrient metabolism. Our previous study showed that iron supplementation (>1 g ferric iron/kg diet) increased ER stress responses and impaired insulin signaling pathways in a rat model of streptozotocin/nicotinamide-induced diabetes [17]. ER stress-associated mechanisms are also required for ferritin-mediated macrophage [18] and hepatic stellate cell activation [19]. Vecchi et al. demonstrated that ER stress-associated mechanisms also promote anemia of inflammation (indicated by decreased serum iron and increased hepatic iron accumulation) via directly regulating the expression of the iron-regulatory hormone, hepcidin [20]. In the present study, we investigated the effects of ER stress on RBC rheology and free Hb degradation pathways in a rat model.

## 2. Results

### 2.1. ER Stress Attenuates RBC Aggregability and Free Hb Uptake

**Figure 1:**
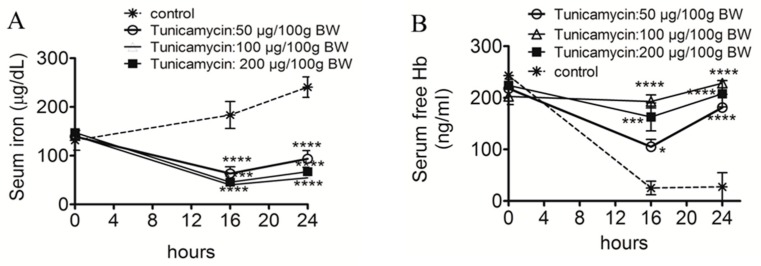

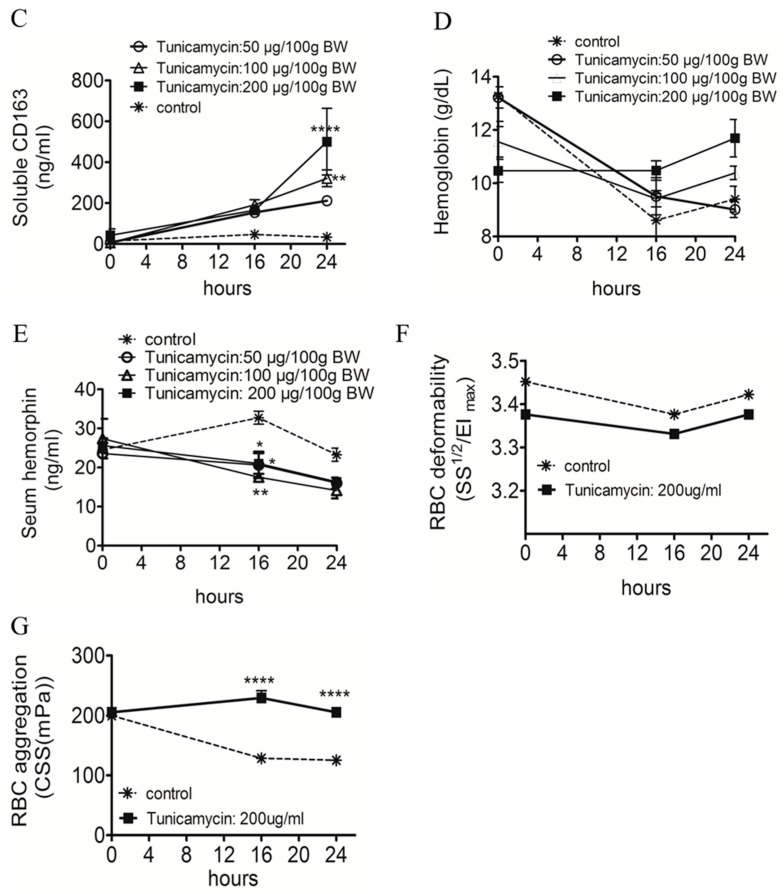
Effects of endoplasmic reticular stress on red blood cells, hemoglobin, and serum iron biomarkers. Rats were injected with various doses of tunicamycin (50, 100, and 200 μg/100 g body weight) for 18 h. Serum levels of iron (**A**), free hemoglobin (**B**), soluble CD163 (**C**), hemoglobin (**D**), and LVV-hemorphin-7 (**E**) levels were quantitated in rats. Red blood cell deformability (**F**) and aggregation (**G**) were measured by microfluidic ektacytometry. A repeated-measures analysis of variance (ANOVA) was employed for the time course analysis (*n* = 4 or 5/group). Significant differences are indicated by * *p* < 0.05, ** *p* < 0.01; *** *p* < 0.001; or **** *p* < 0.0001 compared to the controls at each time point.

We performed animal studies to test whether ER stress-associated mechanisms affected RBC rheology and CD163+ macrophages mediated free Hb uptake and degradation. An animal study showed that a TM injection, a potent ER stress inducer through the inhibition of *N*-glycosylation, significantly decreased serum iron (Figure 1A), attenuated free Hb recycling in the serum (Figure 1B), and increased shedding of the CD163 surface receptor (Figure 1C) compared to control rats. Although no significant difference was observed in Hb levels (Figure 1D), TM reduced the release of LVV-hemorphin-7 (Figure 1E), a peptide derived from the proteolytic degradation of the Hb β chain. TM did not change RBC deformability (Figure 1F), but maintained RBC aggregability (Figure 1G).

### 2.2. ER Stress Alters Hepatic Hepcidin-Ferroportin Expression

**Figure 2:**
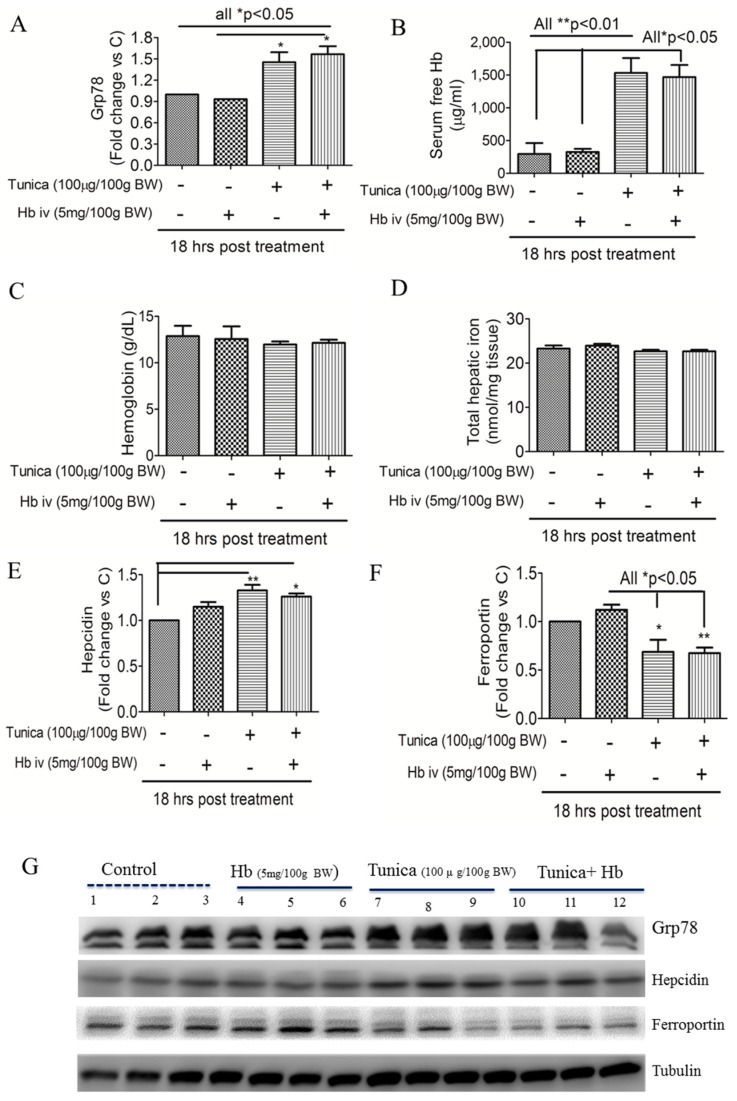
Effects of endoplasmic reticular stress on free hemoglobin and hepatic iron levels. Rats were injected with tunicamycin (100 μg/100 g body weight) for 18 h followed by a hemoglobin (5 mg/100 g body weight) injection. At 10 min after the hemoglobin injection, expression levels of hepatic Grp78 protein (**A**,**G**), serum free hemoglobin (**B**), and hemoglobin (**C**) were measured. Hepatic total iron levels were quantitated by an iron assay kit (**D**). Hepatic expressions of hepcidin (**E**,**G**) and ferroportin (**F**,**G**) were detected by a Western blot analysis. Data are expressed as the mean ± SEM (*n* = 3 per group). * *p* < 0.05; ** *p* < 0.01 vs. the controls or hemoglobin injection by a one-way ANOVA with the Bonferroni posttest and correction.

Figure 2 shows that compared to controls, a TM (100 μg/100 g BW) or TM plus Hb (5 mg/100 g BW) injection significantly increased hepatic Grp78 protein expression (Figure 2A,G) and free Hb (Figure 2B) levels in the serum. Short-term treatment with an ER stress activator did not alter Hb (Figure 2C) or total hepatic iron concentrations (Figure 2D). However, a Western blot analysis showed that an ER stress activator increased hepatic hepcidin (Figure 2E,G) but suppressed ferroportin protein expressions (Figure 2F,G).

### 2.3. ER Stress Suppresses Globin Degradation

**Figure 3:**
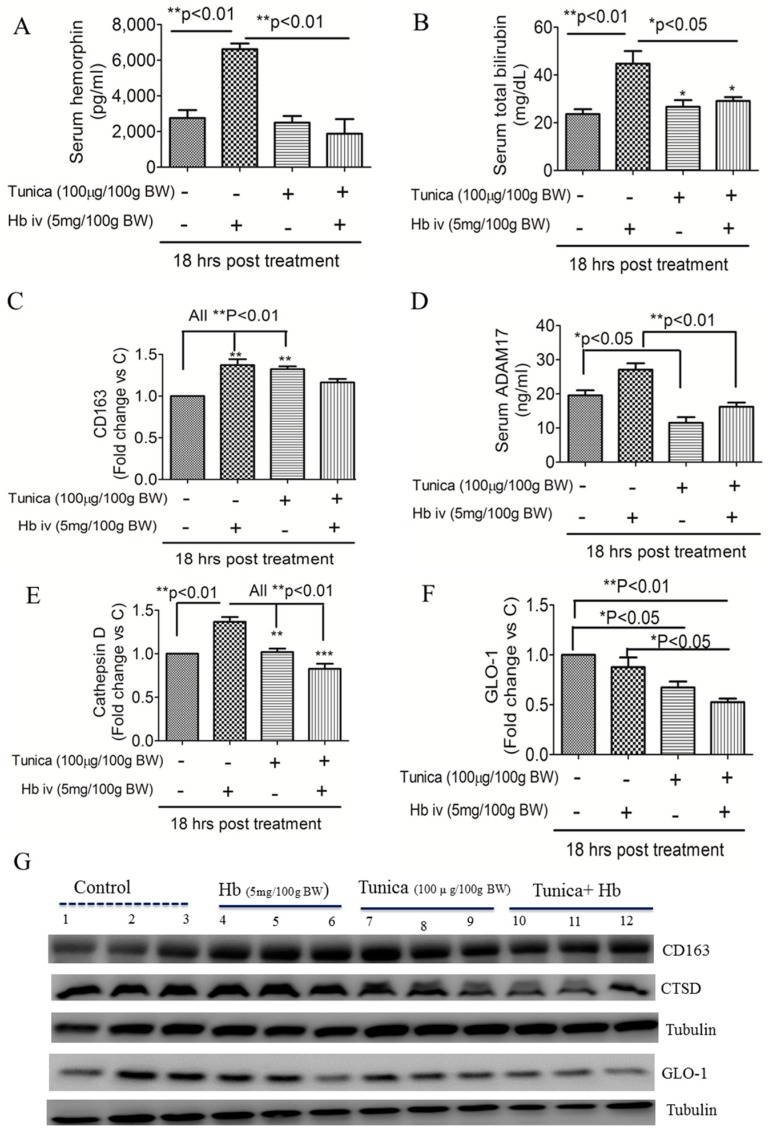
Effects of endoplasmic reticular (ER) stress on hemoglobin degradation protein expressions in the serum and livers of rats. Serum LVV-hemorphin-7 (**A**), total bilirubin (**B**), and ADAM17 levels were measured (**D**). Hepatic expressions of hemoglobin uptake and degradation proteins of CD163 (**C**,**G**), cathepsin D (CTSD) (**E**,**G**), and glyoxalase-1 (GLO-1) (**F**,**G**) were detected by a Western blot analysis (**C**,**D**). Data are expressed as the mean ± SEM (*n* = 3 per group). * *p* < 0.05; ** *p* < 0.01; or *** *p* < 0.001 vs. the controls or hemoglobin injection by a one-way ANOVA with the Bonferroni posttest and correction.

We next investigated how ER stress affected Hb-degradation pathways. Figure 3 shows that, compared with controls, an Hb injection increased serum LVV-hemorphin-7 levels (Figure 3A) and total bilirubin levels (Figure 3B), but this effect was suppressed by TM (all *p* < 0.05). A Western blot analysis showed that Hb or TM injection triggered hepatic CD163 protein expression (Figure 3C,G). An Hb injection also slightly increased serum ADAM17 levels, a metalloproteinase which is known to cleave the full-length CD163 receptor, but an ER stress activator reversed this effect (Figure 3D). The ER stress activator also suppressed Hb degradation proteins (CTSD and GLO1) (Figure 3E–G).

### 2.4. ER Stress Alters Heme Iron Degradation via Increasing Heme Oxygesnase-1 Trafficking

**Figure 4:**
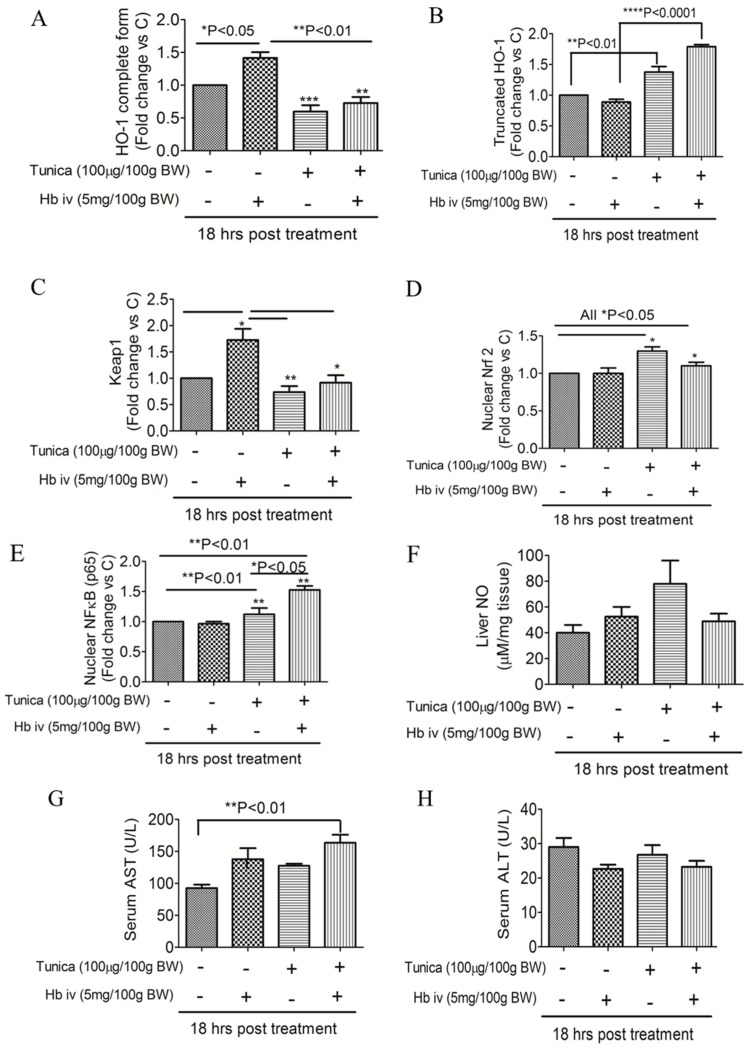

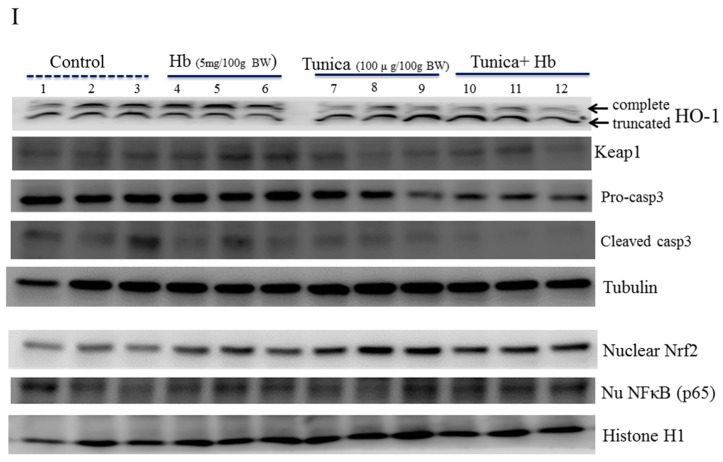
Effects of endoplasmic reticular stress on heme oxygenase (HO)-1, nuclear factor-erythroid 2-related factor 2 (NHrf2), and nuclear factor (NF)-κB (p65) protein expressions in the livers of rats. Expressions of the HO-1 complete form (**A**,**I**) and truncated form (**B**,**I**), Keap-1 (**C**,**I**), nuclear Nrf2 (**D**,**I**), and NF-κB p65 subunits (**E**,**I**) were detected by a Western blot analysis. Hepatic nitrite was detected by the Griess reagent system (**F**). Serum AST (**G**) and ALT (**H**) levels were measured using a colorimetric method. Data are expressed as the mean ± SEM (*n* = 3–4 per group). * *p* < 0.05; ** *p* < 0.01; or *** *p* < 0.001; or **** *p* < 0.0001 vs. the controls or hemoglobin injection by a one-way ANOVA with the Bonferroni posttest and correction.

Figure 4 shows that an Hb injection increased the HO-1 complete form (Figure 4A,I) and keap-1 expression (Figure 4C,I), but this effect was suppressed by TM. In contrast, the ER stress activator increased truncated HO-1 expression (Figure 4B,G), and the translocation of Nrf-2 (Figure 4D,I) and NF-κB (p65) (Figure 4E,I) to nuclei but not cleaved-caspase-3 (Figure 4I). The TM injection increased hepatic NO levels, but this did not reach statistical significance (Figure 4F). There was significant elevation in serum AST levels in rats that received a TM plus Hb injection when compared with controls (Figure 4G). No statistically significant difference was observed in serum ALT levels among groups (Figure 4H).

## 3. Discussion

Liver is the primary organ for iron storage and iron dysfunction can cause liver damage. Our study agrees with Vecchi and colleague’s observation in which that ER stress impairs intracellular iron efflux via altering hepatic hepcidin-ferroportin expression levels [20]. Our study further demonstrated that ER stress-associated mechanisms also affected RBC aggregability and free Hb uptake and degradation. The attenuation of free Hb recycling is likely due to increased shedding of the full-length CD163 receptor on macrophages via ADAM-17-independent pathways. In addition, ER stress responses impair hepatic Hb metabolism through down-regulation of (1) globin degradation (indicated by decreased circulating hemorprotein and globin-degradation proteins CTSD and GLO-1) and (2) heme iron degradation (indicated by decreased keap-1 and HO-1 complete form). Therefore, an inability to recycle and metabolize free Hb may promote liver injury due to the molecular instability of free heme iron, as well as decreased levels of Hb-derived bioactive peptides and byproducts (e.g., LVV-hemorphin-7 and bilirubin).

The sCD163 molecule is regarded as a macrophage activation marker or an acute-phase reactant. Currently, the pathophysiological role of elevated sCD163 in liver injury remains largely unknown. TNF-α-cleaving enzyme (TACE)/ADAM17 and neutrophil elastase are known to cleave surface CD163 receptors on monocytes and macrophages [21,22]. Etzerodt et al. showed that although sCD163 is detectable in mouse serum, shedding of the CD163 receptor in mice is ADAM17-independent [22,23]. Our data agree with this notion. We found that a TM injection increased serum sCD163 levels, but no difference was found in serum ADAM17 levels. This suggests that similar to mice, shedding of the CD163 receptor in rats is independent of ADAM-17. Those authors also suggested that unlike humans, mouse Hb does not have a high affinity for Hp, but binds with high affinity to CD163 [23]. This indicates that the clearance of free Hb may vary depending on the animal species. Nonetheless, our clinical study found that serum sCD163 and free Hb levels were closely associated with fatty liver severity (JS Chang preliminary data). This suggests that under inflammatory conditions or exposure to ER stress, increased shedding of the CD163 receptor may downregulate macrophages’ ability to scavenge free Hb.

After macrophages take up senescent RBCs or free Hb, lysosomal proteolytic enzymes degrade the globin moieties to produce various bioactive peptides [14]. Hemorphins are short peptides derived from the N-terminal region of the β-globin chain that shares a central tetrapeptide core of Tyr-Pro-Trp-Thr [24]. LVV-hemorphin-7, a ten-residue peptide, can decrease blood pressure through inhibiting angiotensin-converting enzymes [25]. LVV-hemorphin-7 also plays important roles in learning and memory [25,26]. Clinical studies showed that serum LVV hemorphin-7 levels were lower in patients with obesity and type 2 diabetes compared to controls [27,28]. Our study showed that an Hb injection increased serum LVV-hemorphin-7 and total bilirubin levels, but this effect was suppressed by the TM injection. This suggests that hemoprotein degradation is also subjected to ER stress.

Our study demonstrated that ER stress may control heme iron degradation via regulating HO-1 trafficking, the key enzyme involved in heme degradation. HO-1 is a smooth ER-anchored protein that faces the cytosol [29]. When heme is released from hemoprotein, HO-1 couples with cytochrome p450 reductase to degrade heme iron into ferrous iron and generate bilirubin and CO as byproducts. However, Lin and colleagues indicated an alternative role of HO-1 [30]. Lin et al. first reported the translocation of HO-1 to nuclei, and nuclear HO-1 may activate genes that promote cytoprotection against oxidative stress [30]. Further study demonstrated that nuclear HO-1 partners with Nrf2, and the HO-1-Nrf2 interaction triggers antioxidative stress responses [31]. Our study showed that an Hb injection increased expressions of the HO-1 complete form and keap-1, but this effect was suppressed by TM. In contrast, an ER stress activator increased truncated HO-1 expression and translocation of Nrf-2 and NF-κB (p65) to nuclei. This suggests that under ER stress conditions, HO-1 and Nrf-2 may be translocated to nuclei to alleviate oxidative stress. Nuclear HO-1-Nrf-2 is also known to activate ferroportin expression, which may help release recycled iron to peripheral areas and prevent tissue iron accumulation. Our data showed that TM increased nuclear Nrf-2 but decreased ferroportin, suggesting that Nrf-2-independent pathways may be involved in downregulating ferroportin. Our study also raised the possibility that increased HO-1 isoforms may impair the HO-1 enzymatic ability to convert heme iron under ER stress.

There are several commercially available pharmaceutical ER stress inducers including tunicamycin and thapsigargin and brefedin A [32]. Brefedin A inhibits the transport of protein from the ER to Golgi apparatus and this leads to the accumulation of unfolded protein in the ER. Thapsigargin, an inhibitor of sarco/ER Ca^2+^ ATPase, decreases calcium levels and affects the activity of calcium-dependent chaperone in ER. Tunicamycin is an inhibitor of N-linked glycoprotein biosynthesis in the ER. ER stress activators cause misfolded and unfolded protein to become accumulated in the lumen of ER, leading to the activation of the unfolded protein response (UPR) or ER stress response [32]. Our study used tunicamycin to induce ER stress because N-linked glycosylation is known to affect iron acquisition protein (e.g., transferrin, transferrin receptor 2) and iron storage protein (e.g., ferritin) [33,34]. Glycosylation may also affect the iron binding capacity of transferrin and ferritin as alterations in the protein structure may affect protein stability and immunogenicity [34].

ER stress can be induced within a few hours after treatment with ER stress inducers and a longer exposure time tends to trigger cell death [32]. Our study showed that 18 h treatment of tunicamycin or tunicamycin plus Hb injection did not cause elevation in levels of serum ALT, hepatic NO, and pro-and cleaved caspase 3 protein. This suggests that the liver is able to recover from acute ER stress and a longer treatment time may be necessary to see the tunicamysin-mediated liver injury or cell death. Another possibility is that caspase-3 may not be sensitive enough to reflect ER-tress mediated apoptosis. The literature shows that ER stress mediated cell death is C/EBP homologous protein (CHOP) dependent [35]. The dissociation of ER-resident chaperone *Grp78* from the membrane anchored receptors ATF6, IRE1, and PERK triggers three UPR signal transduction pathways: *ATF6, IRE1-XBP1*, and *PERK-eIF2α-ATF4*. Activation of the *PERK-eIF2α* pathway attenuates global protein translation, but it also leads to a selective translation such as apoptotic genes (e.g., *CHOP*, *ATF4*) and anti-oxidative genes (e.g., *Nrf2*) [36]. Our study did not measure *CHOP* due to budget restraints. The present study also found that TM plus Hb injection significantly induced serum AST levels. Serum AST and ALT levels are biomarkers of hepatocellular injury which are released into the blood by the damaged hepatocyte. The ALT enzyme is secreted predominantly in the liver, whilst the AST enzyme can be derived from various organs including the liver, skeletal muscle, kidney, and heart. Collectively, the present results seem to suggest that the UPR is able to alleviate the acute ER stress induced by the short-term exposure of tunicamycin or tunicamycin plus Hb injection. However, 18 h treatment of tunicamycin plus Hb injection may also cause cell death in other organs (e.g., skeletal muscle), as indicated by the increased levels of serum AST.

## 4. Materials and Methods

### 4.1. Rat Experiment

Thirty-Two male Sprague-Dawley (SD) rats aged 10 weeks (374.2 ± 20.0 g) were purchased from BioLasco (Taipei, Taiwan) and kept under standard conditions at the animal facility of Taipei Medical University. The reason that only adult male SD rats were used was to minimize the effects of growth or sex hormone (e.g., estradiol) and menstrual cycle on iron status [37]. The experimental protocol was approved by the Animal Ethical Committee of Taipei Medical University (LAC-2015-0201; (15, 12, 2015)). Tunicamycin (TM) is an inhibitor of *N*-glycosylation and a potent ER stress inducer. For the TM titration experiment (*n* = 4 or 5 rats per group), SD rats were given an intraperitoneal (IP) injection of various doses of TM (50, 100, and 200 μg/100 g body weight (BW)) or vehicle (5% DMSO) to induce acute ER stress. Blood was taken at 0, 16, and 24 h post-injection. In the second experiment, TM (100 μg/100 g BW) was IP-injected into rats at 18 h prior to an intravenous (IV) Hb injection (5 mg/100 g BW) (all purchased from Sigma-Aldrich, St. Louis, MO, USA). Rats were killed at 15 min after the Hb injection.

### 4.2. Blood Biochemistry

Fasting blood samples were collected and separated into RBCs, serum, and plasma. Samples were stored at −80 °C until being analyzed. RBC deformability and aggregation were measured by a microfluidic ektacytometer (RheoScan-AnD 300, RheoMeditech, Seoul, Korea). RBC rheology was defined as either the critical shear stress (CSS) or shear stress required for one-half maximal elongation (SS^1/2^). Greater CSS values reflect higher RBC aggregability [38], and higher SS^1/2^ values indicate a decrease in RBC deformability [39]. Free Hb (ICL; Mountainside, NJ, USA) and soluble CD163 (R&D Systems, Shanghai, China) levels in serum were determined by enzyme-linked immunosorbent assay (ELISA) kits, according to the manufacturer’s instructions. Hb levels were assessed by a colorimetric method (Fortress Diagnostics, Northern Ireland, UK). Serum total bilirubin was quantitated with a Bilirubin Assay Kit (Cell Biolabs, San Diego, CA, USA) based on the Jendrassik-Frof method. Serum and liver total iron (40 mg liver sample/test) were quantitated by an iron assay kit (Abcam, Cambridge, UK) according to the manufacturer’s instructions. Serum levels of aspartate aminotransferase (AST) and alanine aminotransferease (ALT) were measured using a colorimetric method by the Beckman DxC 800 (Beckman Coulter, Brea, CA, USA).

### 4.3. Western Blot Analysis

Total protein was extracted from whole-liver tissues as previously described [17]. Briefly, 50 mg of tissues was lysed in RIPA buffer, and the protein concentration was determined with a Pierce™ BCA Protein Assay Kit (Thermo Fisher Scientific, Waltham, MA, USA). Nuclear and cytosolic fractions were separated from the liver lysates using an NE-PER Nuclear and Cytoplasmic Extraction kit (Thermo Fisher Scientific, Taipei, Taiwan). The nuclear *Nrf2* and *NFκB (p65)* were normalized against Histone H1 (Figure 4G). Anti-rat antibodies (Abs) for *tubulin*, *histone*, *CTSD*, *GLO-1*, *GRP78*, *hepcidin*, *ferroportin* (FPN), *nuclear factor (erythroid-derived 2)-like 2* (Nrf2), *keap1*, *CD163*, *HO-1*, *caspase-3*, and *nuclear factor (NF)-κB (p65)* were used at dilutions of 1:1000 or 1:2000 to detect immunoreactive signals. Antibodies were purchased from Santa Cruz Biotechnology (Dallas, TX, USA), except for *Grp78* (Cell Signaling, Danvers, MA, USA), *GLO-1* (Abcam, Cambridge, UK), *CTSD* (Sigma-Aldrich, St. Louis, MO, USA), *histone H1*, *caspase-3* (GeneTex, Irvine, CA, USA), and *HO-1* (Enzo Life Sciences, Lausen, Switzerland).

### 4.4. Statistical Analysis

Statistical analyses were performed using GraphPad Prism 5 (La Jolla, CA, USA). A repeated-measures analysis of variance (ANOVA) was employed for the time course analysis. The trend test was analyzed by a general linear model for continuous variables. A one-way ANOVA with the Bonferroni posttest and correction was used to examine the means of more than two groups. Data are presented as the mean ± standard error of the mean (SEM). *p* < 0.05 was considered statistically significant.

## 5. Conclusions

Successful Hb metabolism not only protects cells against free Hb-mediated toxicity, but Hb degradation byproducts (e.g., LVV-hemorphin-7, CO, and biliverdin) also exert important anti-inflammatory effects [14]. Regulation of Hb metabolism by ER stress further strengthens the important role of ER stress in controlling iron metabolism under conditions of cellular stress.
